# Green Extraction of Polyphenols via Deep Eutectic Solvents and Assisted Technologies from Agri-Food By-Products

**DOI:** 10.3390/molecules28196852

**Published:** 2023-09-28

**Authors:** Man Zhou, Olugbenga Abiola Fakayode, Haoxin Li

**Affiliations:** 1 School of Food and Biological Engineering, Jiangsu University, Zhenjiang 212013, China; fakayode@ualberta.ca (O.A.F.);; 2 Department of Mechanical Engineering, University of Alberta, Edmonton, AB T6G 2R3, Canada

**Keywords:** polyphenols, deep eutectic solvents, green extraction mechanism, assisted technologies, agri-food by-products

## Abstract

Polyphenols are the largest group of phytochemicals with important biological properties. Their presence in conveniently available low-cost sources, such as agri-food by-products, has gained considerable attention in their recovery and further exploitation. Retrieving polyphenols in a green and sustainable way is crucial. Recently, deep eutectic solvents (DESs) have been identified as a safe and environmentally benign medium capable of extracting polyphenols efficiently. This review encompasses the current knowledge and applications of DESs and assisted technologies to extract polyphenols from agri-food by-products. Particular attention has been paid to fundamental mechanisms and potential applications in the food, cosmetic, and pharmaceutical industries. In this way, DESs and DESs-assisted with advanced techniques offer promising opportunities to recover polyphenols from agri-food by-products efficiently, contributing to a circular and sustainable economy.

## 1. Introduction

The agricultural and food industries generate large amounts of by-products throughout the food supply chain. The Food and Agricultural Organization (FAO) estimates that 1.3 billion tons of food are lost or wasted globally each year [1]. The disposal of these agri-food by-products costs food manufacturers a lot and has adverse impacts on the environment. On the other hand, these by-products are abundant, low-cost, and renewable sources of high-value-added compounds. Valorizing agri-food by-products into high-value-added compounds provides a unique roadmap for realizing the Sustainable Development Goals (SDGs) 12 (Responsible Consumption and Production) of the United Nations. Among the high-value-added compounds, polyphenols have attracted increasing attention due to their multiple health benefits.

**Figure 1:**
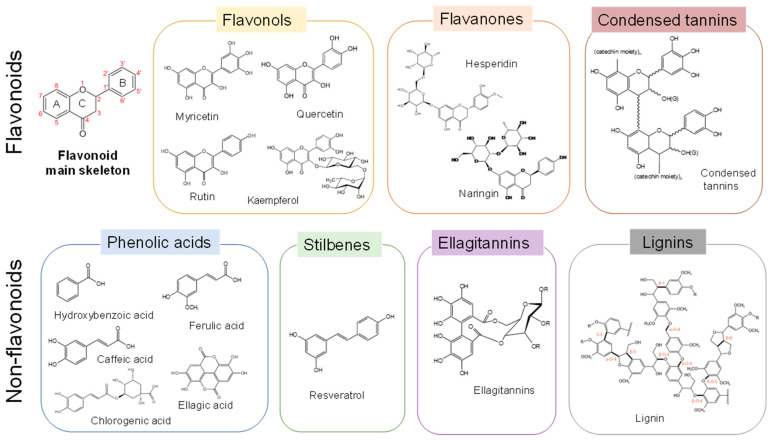
Classification and structure of polyphenols.

Polyphenols are broadly present in the plant kingdom and play various important roles in growth and development processes. The protective role of polyphenols against radical oxygen and reactive nitrogen species, UV light, and plant pathogens results in a variety of beneficial bioactivities, including antioxidant, antimicrobial, anticarcinogenic, and antidiabetic activities [2]. Polyphenols have one or more aromatic rings carrying one or more hydroxyl groups. From the chemical structure standpoint, as shown in Figure 1, polyphenols are classified into two subgroups: flavonoids and nonflavonoids [3]. Flavonoids, including flavonols, flavanones, and condensed tannins, are built around a C6-C3-C6 carbon skeleton. Nonflavonoids include phenolic acids, stilbenes, gallotannins, ellagitannins, and lignins.

The efficient extraction of polyphenols from various agri-food by-products is a preliminary and pivotal step. The most common strategies are solid–liquid extraction (SLE) and liquid−liquid extraction. The traditionally used solvents, such as acetone, methanol, ethanol, or their mixtures, are organic. Traditional solvents no longer meet green and sustainable development due to drawbacks such as high costs, difficult synthesis techniques, high toxicity, and poor recoverability [4]. With the rise of green and sustainable techniques, researchers are focusing their efforts on searching for new alternative solvents to replace traditional media. Deep eutectic solvents (DESs) have recently acquired popularity for extracting and valorizing polyphenols due to its superior ability to solubilize polyphenols [3,4,5]. DESs align with the 12 principles of green chemistry and has many remarkable advantages, such as low cost, ease of synthesis, tunable characteristics, good recyclability, and high biodegradability [6]. DESs are claimed to be green and safe, especially for natural DESs (NADESs) [4,7,8]. NADESs are composed of naturally occurring components and are generally recognized as safe (GRAS) [4]. The use of NADESs is allowed in food and pharmaceutical formulations [8].

Given the escalating demand for polyphenols for advanced industrial applications, this review provides a compilation of the newest information on the green extraction of polyphenols using DESs and advanced assisted technologies. It focuses on the recent advances in polyphenol-rich agri-food by-products, polyphenols extraction using DESs, improvement of extraction via assisted technologies, and applications in the food, cosmetic, and pharmaceutical industries. In addition, the future perspectives for addressing the challenges of polyphenol extraction using DESs are presented.

## 2. Polyphenol-Rich Agri-Food By-Products

A wide range of agri-food by-products are rich in polyphenols. Much research has been conducted to extract phenolic compounds from these agri-food by-products using DESs, including fruit by-products, vegetable by-products, tree nut by-products, cereal by-products, oil-bearing crop by-products, and other agri-food by-products.

### 2.1. Fruit By-Products

Fruits have a crucial role in our daily diet. Due to their perishable nature, fresh fruits are processed into various products. During the industrial process, tremendous amounts of by-products and waste are generated. These by-products are rich in different valuable compounds. Table 1 lists some recent applications of DESs and assisted techniques for extracting polyphenols from fruit by-products.

Apple is one of the largest fruit producers in the world. The major by-product generated during the apple juicing process is apple pomace. It is comprised of peels and flesh (90%), seeds (2~4%), and stems (1%), and these by-products are rich in polyphenols. It has been reported that DESs can efficiently extract quercetin glycosides, kaempferol, catechin, and procyanidins from apple pomace [9,10,53]. Extracts rich in these compounds exhibit cardioprotective, anticancer, and antimicrobial biological activities.

Citrus fruits, such as orange, mandarin, lemon, grapefruit, pomelo, lime, etc., are widely grown and consumed across the globe. Tremendous by-products are produced during the industrial production of juice, jellies, candied fruits, and jams. Various flavonoids (e.g., hesperidin and naringin) and phenolic acids have been recovered from citrus by-products using DESs [11,12,13,14,15,16]. For instance, naringin can be used as a food antioxidant or ingredient to treat obesity and diabetes. In addition, these compounds can be used as food additives to impart bitter taste [54].

Grape is one of the most widely cultivated fruits. According to the statistical data of FAO, the annual production of grapes is up to 75 million tons, and about 80% is used to manufacture wines and their derivatives. Consequently, a large quantity of grape by-products is globally produced each year. The principal by-product of the grape wine industry is grape pomace, consisting of residual pulp, skins, stems, and seeds. Grape pomace is a good source of polyphenols such as lignin, condensed tannins (proanthocyanidins), anthocyanins (e.g., malvidin-3-O-monoglucoside), phenolic acids, catechin and epicatechin derivatives, and hydroxytyrosol. These compounds possess various health benefits, including antimicrobial, anti-inflammatory, anticarcinogenic, cardioprotective, and neuroprotective activities [54].

Various berries, including blueberry, cranberry, raspberry, mulberry, sea buckthorn berry, and sour cherry, are used to manufacture juice, wine, and derivative products to extend shelf-life and economic value chain. Consequently, millions of tons of berry by-products are annually generated in the world. These berry by-products comprise leaves and pomace during harvest, juicing, and fermentation and are rich sources of phenolic compounds, especially anthocyanins. ChCl:malic acid efficiently extracted anthocyanins from Brazilian berries, and the anthocyanin-rich extracts showed promising antidiabetic and anti-obesity potential [26]. By using DESs, plenty of anthocyanins have been recovered from grape skin [17,18,19,20], grape pomace [21,22,23,24], red wine lees [25], jaboticaba pomace [27], blueberry pomace [26,28,29,30], blueberry wine residues [31], and other berries pomace [32,33,34,35,36].

Other fruits, such as pear, peach, pomegranate, mango, mangosteen, and date, are widely grown and much-consumed fruits. They can be consumed as fresh fruit or processed products. Industrial processing results in huge amounts of by-products; for example, the processing of pomegranate juice generates high quantities of peels and seeds. Various phenolic compounds, for example, ellagitannins, have been extracted using DESs from pomegranate peels, mesocarps, and seeds [37,38,39,40,41]. Mangiferin is the prominent phenolic compound in the DESs extract from mango peel, which showed excellent antioxidant capacity to protect sunflower oil from oxidation [42].

### 2.2. Vegetable By-Products

Onion, potato, and tomato are important vegetables that can be processed into various dehydrated, powdered, fried, or canned products. Consequently, huge amounts of by-products are produced during their industrial processing. These by-products are mainly comprised of peel, skin, and pomace. As shown in Table 2, they are excellent natural resources for many valuable polyphenols. Onion solid by-products contain high amounts of flavonoids such as quercetin, kaempferol, and myricetin [55,56,57,58,59,60]. Quercetin is a naturally occurring flavonoid that has cardioprotective, neuroprotective, anticoagulant, anti-inflammatory, anticarcinogenic, and antioxidant activities [60]. It has been reported that the NADESs extract from onion peel showed higher antioxidant capacity than standard ascorbic acid [57]. Tomato is rich in flavones like chlorogenic acid, rutin, and quercetin [44,61]. Numerous phenolic compounds have been extracted from onion and tomato by-products using DESs, such as quercetin [58,60], kaempferol glycosides [59], and anthocyanins [62]. Additionally, DESs show high extraction efficiency in retrieving phenolic compounds from other vegetable by-products, like kale waste [63], bitter melon leaves [64], pepper leaves [65], and lotus leaves [66].

### 2.3. Tree Nut By-Products

In the last decades, the consumption of tree nuts (such as *Carya cathayensis* Sarg, chestnut, and hazelnut) has boosted sharply worldwide due to their nutritional worth and health benefits. Their industrial processing generates vast amounts of underexploited by-products enriched in high-valued phenolic compounds, like ellagic acid, gallic acid, catechin hydrate, procyanidin, and myricetrin. As listed in Table 3, ever-growing studies have successfully used DESs to extract various phenolic compounds from these tree nut by-products [67,68,69,70,71]. For example, ChCl:malic acid extracted 20 phytochemicals from *Carya cathayensis* Sarg. peels, majorly including catechin, procyanidin B1 and B3, 2,3-dihydroxybenzoic acid, pinocembrin, and myricetrin. Additionally, the NADESs extract possessed the maximum content of phenolic compounds and antioxidant activity, as well as α-glucosidase and α-amylase inhibitory effects [70]. A high content of ellagic acid (4.64 mg/g) with high purity (85.6%) can be extracted using ChCl:n-propanol from chestnut shells [68].

### 2.4. Cereal By-Products

Wheat, rice, foxtail millet, and buckwheat are important and highly consumed cereals worldwide. They serve as very efficient and major sources of human energy and nutrition. The industrial processing (like milling and brewing) of cereals produces enormous amounts of bran, husk, hull, and spent grain. For example, about 35 million tons of wet brewer spent grain are produced annually during the worldwide manufacture of beer [72]. As shown in Table 4, these by-products are major sources of natural rutin, flavonoids, and anthocyanins. As a green and sustainable extraction medium, DESs have extracted polyphenols from wheat barn [73] and brewer spent grain [72], anthocyanin from black rice bran [74], and rutin from tartary buckwheat hull [75]. The polyphenol-rich NADESs extract from foxtail millet bran was mainly composed of coumaric acid, apigenin-C-dihexoside, and *p*-coumaroylspermidine, and showed high acetylcholinesterase inhibitory activity [76].

### 2.5. Oil-Bearing Crop By-Products

Olive, soybean, peanut, sunflower, and rapeseed are the primary oil-bearing crops in the world. Most of them are used to produce edible vegetable oils via physical pressing or solvent extraction. During these industrial processes, very large quantities of by-products are produced, including olive oil pomace, olive tree leaves, sunflower disks, and peanut hulls. As listed in Table 5, oleuropein, flavones (luteolin-7-glucoside and luteolin), isoflavone (daidzein, genistein, and puerarin), flavonols (rutin and quercetin), flavan-3-ols (catechin), phenolic acids (chlorogenic acid), and substituted phenols (hydroxytyrosol) are rich in these by-products [78,79,80,81,82,83,84,85,86]. One of the most bioactive molecules possessing anti-inflammatory and antiplatelet effects is hydroxytyrosol [80,87,88,89]. Another bioactive molecule with anti-hyperglycemia, cholesterol-lowering, anticarcinogenic, antidiabetic, and antiallergic activities is isoflavone. They have been efficiently extracted using DESs from soy molasses and kudzu roots [82].

### 2.6. Other Agri-Food By-Products

Other agri-food by-products, such as cocoa and coffee, are also excellent resources for polyphenols. The coffee production chain generally consists of eight process units: planting, harvesting the cocoa beans, drying, milling, tasting, roasting, grinding, and brewing. Approximately 10.5 million tons of cocoa by-products were generated globally during the 2020/21 season [90]. As shown in Table 6, procyanidins and chlorogenic acids have been efficiently extracted from cocoa by-products [91,92] and spent coffee grounds [93,94] using various DESs as the extraction media. In addition, xanthohumol, which possesses a variety of bioactivities, has been sustainably and simply recovered from spent hops using ChCl-based DESs [95].

## 3. Use of DESs as Green Solvents in the Extraction of Polyphenols from Agri-Food By-Products

### 3.1. DESs and Its Mechanism of Polyphenol Extraction

**Figure 2:**
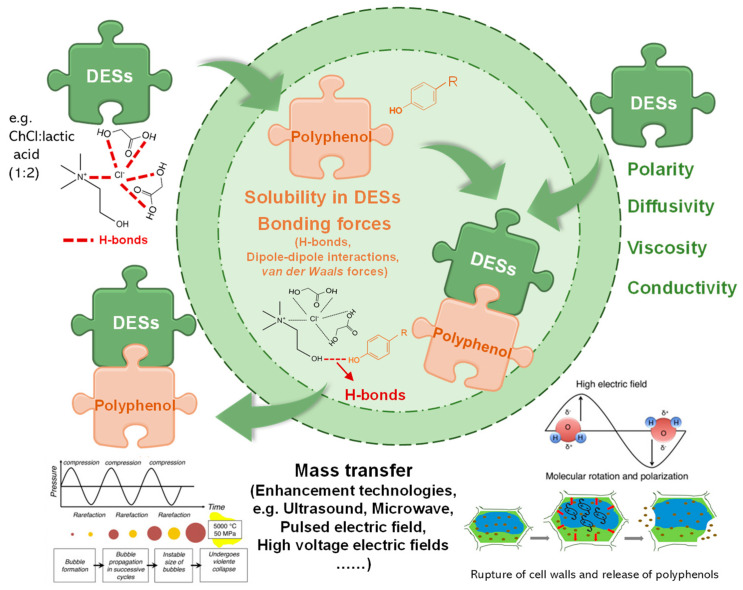
Simplified mechanisms of polyphenol dissolution in DESs media.

DESs are liquid eutectic mixtures of hydrogen bond acceptors (HBAs) and hydrogen bond donors (HBDs) (Figure 2). The establishment of hydrogen bonds (H-bonds) results in the formation of a homogeneous eutectic mixture after a simple stir of these components under mild conditions. DESs are becoming increasingly important in the agri-food industry for producing clean-labeled products that consumers demand [7]. DESs can be prepared using a wide range of chemicals. Choline chloride (ChCl) usually functions as the HBA, while carbohydrates, alcohols, acids, amides, and phenolic compounds function as HBDs. As shown in Table 1, Table 2, Table 3, Table 4, Table 5 and Table 6, ChCl-based DESs are the optimum solvents for efficiently extracting polyphenols. In general, the polarity, diffusivity, viscosity, and conductivity are crucial physicochemical properties of DESs that influence the extraction yield of polyphenols. This review does not cover the physicochemical properties of DESs, as readers are advised to refer to the related content in our previous study [6].

The extraction of polyphenol molecules from agri-food by-products can be considered an SLE process [96]. According to the principle of SLE, the extraction process of polyphenols using DESs includes mass transfer from a solid to a liquid phase and polyphenol dissolution in DESs (as shown in Figure 2). DESs are good solvents with rare solvation properties [3] and can highly solvate polyphenol due to its similar polarities and chemical interactions, such as H-bonds, dipole–dipole interactions, and van der Waals forces. The H-bonds interactions between DESs and polyphenol are so strong (stronger than water solute) that they outweigh the other polyphenol−polyphenol electrostatic forces. The van der Waals interactions become weaker than the steric hindrance of the ether groups, resulting in aromatic ring dispersion in DESs. The high dissolution of polyphenols and diffusivity of DESs facilitate polyphenol molecules’ diffusion outside of plant cells. The affinity between polyphenols and DESs can be theoretically elucidated via a conductor-like screening model for real solvents (COSMO-RS) analysis [15,21,27,87].

**Figure 3:**
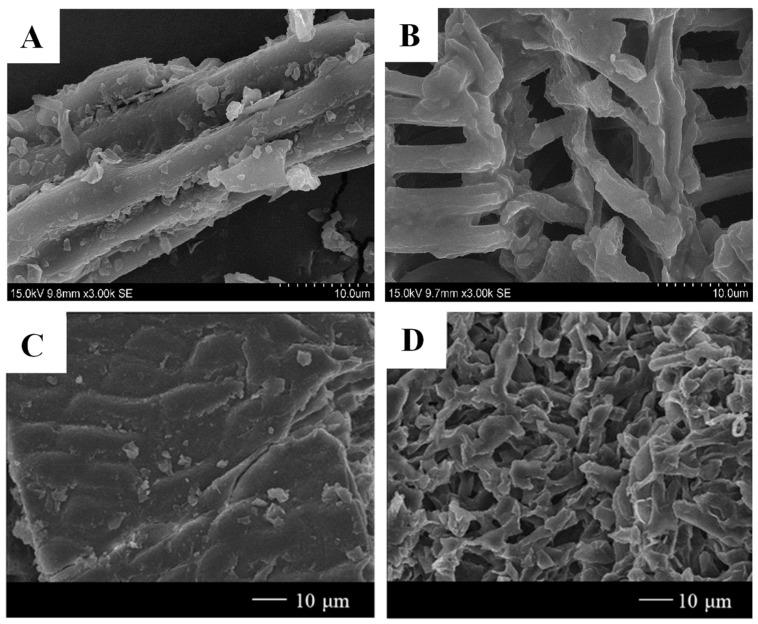
SEM images of mulberry leaves before DESs extraction (**A**) and after DESs extraction (**B**) and FE-SEM images of *Pyrus ussuriensis* leaves surface before DESs extraction (**C**) and after DESs extraction (**D**) (reproduced from [35,45] with permissions from Elsevier license number 1400088-1).

The effect of DESs on the cell structure of agri-food by-products can be intuitively revealed via scanning electron microscopy (SEM) and field emission SEM (FE-SEM). SEM images of orange peel cell structures after ChCl-based DESs extraction showed a higher disintegration level than raw material, suggesting that ChCl-based DESs were efficient for the dissolution of cell wall structure [16]. Figure 3A,B show the microstructure of mulberry leaves before and after extraction with DESs using SEM [35]. As shown in Figure 3A, the cell wall structure showed integrity before extraction; however, when treated with ChCl:citric acid, the cell walls were entirely damaged (Figure 3B). A SEM image of ChCl:MA soaked *Carya cathayensis* Sarg. peels exhibited rough and rugae structures on its outer surface, which was ascribed to the partial erosion and penetration of DESs on the cell wall [69]. More pronounced damage was observed for olive leaves with DESs than ethanol [86]. The FE-SEM images of the pre- and post-extraction *Pyrus ussuriensis* leaves are shown in Figure 3C,D [45]. As can be observed in Figure 3C, there was no solvent penetration on the surface of the *Pyrus ussuriensis* leaves before extraction. However, sufficient solvent penetrations were observed in the FE-SEM images of *Pyrus ussuriensis* leaves after ChCl:glutaric acid extraction (Figure 3D). The high solubility of polyphenols in the DESs facilitated the penetration of the solvent, which led to structural changes in the overall leaf surface.

Furthermore, mass transfer and kinetic studies corroboratively elucidate the extraction mechanism of polyphenols using DESs. A mass transfer result confirmed an efficient contact between polyphenols and DESs according to the high Biot number values [47]. Fick’s model successfully forecasted the extraction kinetics of tannic acid using an ultrasound-assisted DESs extraction method and revealed that diffusivity was the controlling factor [57]. Kinetic studies showed that the extraction diffusivity of anthocyanin in NADESs (1.063 × 10^−12^) was markedly higher than in water (0.835 × 10^−12^) [74]. Moreover, it has been reported that DESs extracted more polyphenols with higher molecular weights and more diverse phenolic compounds than methanol from mangosteen peel [43]. Quantum chemical calculation combined with molecular dynamic simulation revealed that the high extraction efficiency of ChCl:malic acid was due to the large solvent accessible surface area, long lifetime of H-bonds between ChCl:malic acid and extract, and low intermolecular interaction energy [70]. In summary, DESs can serve as an efficient solvent for the extraction of polyphenols.

### 3.2. Process for the Green Extraction of Polyphenols

**Figure 4:**
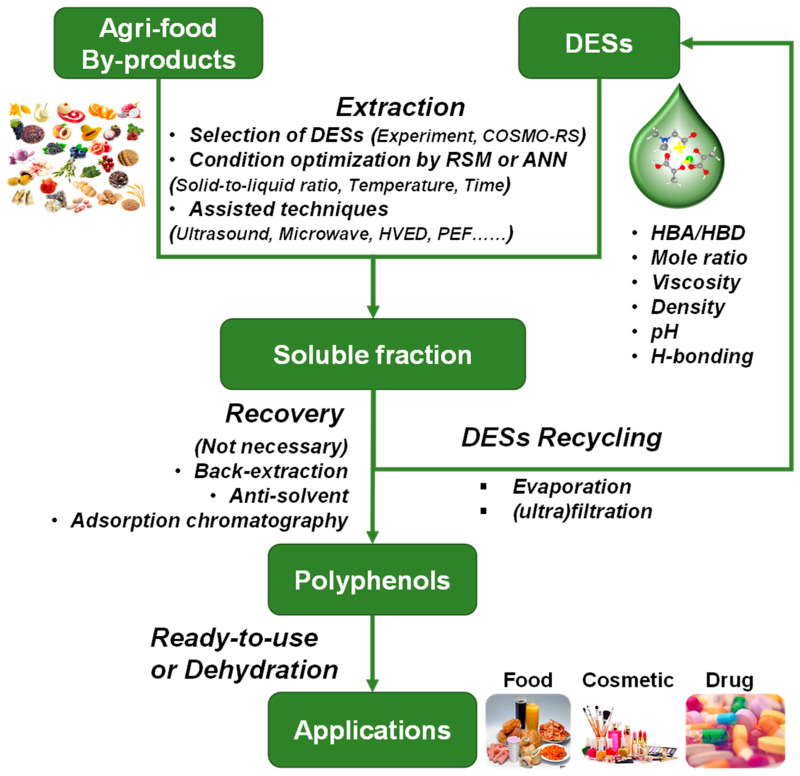
Schematic process for the green and sustainable extraction of polyphenols from agri-food by-products.

In recent years, numerous research studies involved in extracting polyphenols using various DESs have emerged (Table 1, Table 2, Table 3, Table 4, Table 5 and Table 6). The extraction yield of polyphenols is usually determined by the total phenolic content (TPC), which is expressed as a mg gallic acid equivalent (GAE) per gram of dry weight. In addition, the extraction yield of flavonoid and anthocyanin is determined by total flavonoid content (TFC) and total anthocyanin content (TAC), respectively. The composition of phenolic compounds is commonly measured by HPLC-DAD, HPLC-PDA, or HPLC-MS. The higher polyphenol yield of DESs than conventional organic solvents suggests that DESs are efficient solvents for extracting polyphenols. Figure 4 shows the schematic process for the green extraction of polyphenols from agri-food by-products.

The milled agri-food by-products and selected DESs are mixed at a certain solid-to-liquid ratio. Then, the mixture is heated to the preset temperature in a specific device and maintained for a certain time. The selection of the optimum solvent for phenolic compounds is a crucial step that can be achieved using experimental methods or theoretical simulations. COSMOtherm is an in silico approach based on COSMO-RS and can predict the solubility of the target compound in a wide range of solvents [15,21,27,87]. Compared to the experimental method, COSMOtherm is much more time- and labor-saving. In general, the chemical nature of HBAs and HBDs, their molar ratio, viscosity, density, pH, and H-bonding network in the DESs are crucial factors that influence the extraction yield of polyphenols. The extraction conditions (namely solid-to-liquid ratio, water content, temperature and time, and technical parameters of assisted technologies) are usually optimized to maximize the extraction yield of polyphenols. The optimization process can be experimentally conducted and optimized using the response surface method (RSM) and/or artificial neural network (ANN) methods [32,74].

After the extraction process, the resultant polyphenol-rich liquid is taken out to recover polyphenols using various strategies, including back-extraction [13], the addition of anti-solvent [75], and adsorption chromatography with microporous resins [49]. However, in some cases, such as when using NADESs as the extraction medium, the recovery process is not necessary. Furthermore, NADESs can be used as a storage system to stabilize polyphenols or as a formulation system to deliver polyphenols distribution in drug and cosmetic products [96]. At the same time, the DESs-rich supernatant is collected and recycled through anti-solvent evaporation or liquid phase (ultra)filtration. It has been reported that the recycled DESs still maintains an excellent performance in extracting polyphenols with acceptable efficiency [35]. Finally, the recovered polyphenol is ready to use [24,91] or dried into a final product. In summary, this scheme improves the extraction yield of polyphenols, reduces the generation of waste, minimizes the use of chemicals, and paves the way for a sustainable and circular bio-economy.

## 4. Assisted Technologies of Polyphenols Extraction with DESs

Apart from the proper selection of DESs, the extraction process can be improved using assisted technologies. As listed in Table 1, Table 2, Table 3, Table 4, Table 5 and Table 6, various techniques, including microwave, ultrasound, pulsed electric field (PEF), high-voltage electric discharge (HVED), and infrared, have already been combined with DESs to improve the extraction yield of polyphenols. Additionally, the joint use of these assisted techniques and DESs can shorten the extraction time, reduce solvent consumption, and curtail the operation cost.

### 4.1. Ultrasound

Ultrasound is the mostly-used and simplest assisted extraction technique due to the simple requirement of common equipment—ultrasonic bath. Ultrasound-assisted extraction (UAE) is based on the cavitation process generated by compression and rarefaction cycles related to the propagation of ultrasounds through the by-products [54]. As shown in Figure 2, micro-bubbles cavitation, microjets shooting on the surface, and severe agitation caused by mechano-acoustic effects during ultrasonication enhance the micro-pores for greater surface area contact with DESs [6]. As listed in Table 1, Table 2, Table 3, Table 4, Table 5 and Table 6, UAE has efficiently extracted various polyphenols from a wide range of agri-food by-products.

**Figure 5:**
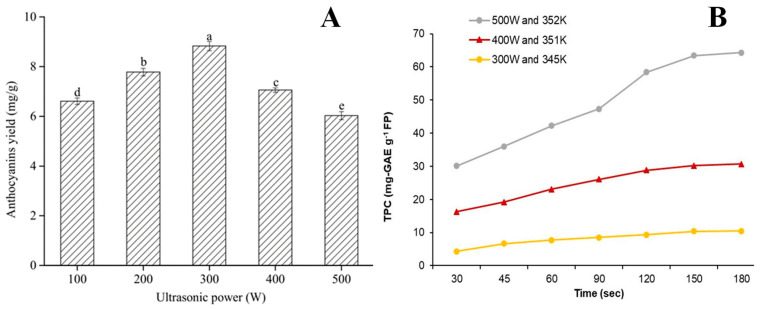
Effects of ultrasonic power on anthocyanins yield (**A**) and microwave power TPC (**B**) (taken from Refs. [31,47] with permission of Wiley, permissions license ID number: 1400450-1). Note: Different lowercase letters indicate significant differences between groups, *p* < 0.05.

Ultrasound power [25,29,30,31,39,76], intensity [10], duty cycle [10,57], and amplitude [43,74] are critical parameters that influence the extraction efficiency. The ultrasonic power significantly affected the extraction yield of anthocyanins, whose content increased with increased ultrasonic power [25]. As shown in Figure 5A, the yield of anthocyanins using DESs was significantly enhanced with increasing ultrasound power and reached the maximum value at 300 W [31], whereas, as the ultrasound power increased above 300 W, anthocyanin yield decreased significantly. A similar trend was observed for the DESs-based UAE of anthocyanins from blueberry pomace [30]. Moreover, similar trends were also reported for the DESs-based UAE of polyphenols from foxtail millet bran [76], apple pomace [10], and pomegranate peel [39]. This was attributed to excessive heat generation of high ultrasound power, which led to the degradation of polyphenols. Rashid and co-workers studied the effect of acoustic intensity on the extraction of polyphenols, and the results showed that increasing the acoustic intensity from 20 to 83.1 W/cm^2^ increased the percentage increase in polyphenols extraction by 60~73.8% for the three tested DESs [10]. This was because of the amplification of ultrasonic waves, which resulted in the intensification of cavitation effect. During the travel of huge amplitude ultrasonic waves via DESs system, higher acoustic intensity led to energetic cavity collapse and shock waves formation. Finally, these actions resulted in interfacial turbulence, outer material disintegration, energy dissipation, and diffusion.

Pulse mode ultrasound is recommended to recover polyphenols as it avoids the cumulative thermal effect during the extraction process. Therefore, the duty cycle of pulse mode ultrasound should be carefully chosen. The extraction yield of tannic acid from onion peel significantly decreased with the increase in the duty cycle [57]. However, a different trend was reported for retrieving polyphenols from apple pomace [10]. The extraction yield of polyphenols increased with increasing duty cycle from 20% to 75%. Additionally, the yield of polyphenols is also affected by ultrasound amplitude. The extraction yield of proanthocyanidin from mangosteen peel was positively affected by ultrasound amplitude [43]. A significant increase in anthocyanin content was observed when raising the amplitude level to 21.25% [74]. However, higher amplitude levels caused a negative effect on anthocyanin content due to the chemical degradation of anthocyanins.

### 4.2. Microwave

The fundamental principle of microwave-assisted extraction (MAE) is dielectric heating [54]. A microwave is a propagating electromagnetic wave that interacts with polar molecules (e.g., DESs). The rotation and polarization of polar molecules in DESs promote the penetration of the DESs into biomass and provide energy to activate the bond-breaking required for the dissolution of polyphenols [6]. MAE intensifies the DESs extraction process as a result of heat and increased mass transfer [54]: (1) penetration of DESs into the biomass matrix; (2) solubilization and/or breakdown of the cellulose, hemicellulose, lignin, and polyphenols; (3) transport of the solubilized compounds from the insoluble biomass matrix to the bulk DESs phase; and (4) separation of the DESs liquid phase and residual solid phase.

As listed in Table 1, Table 2, Table 3, Table 4, Table 5 and Table 6, a high extraction yield of polyphenols has been achieved by synergistically using microwave and DESs from various agri-food by-products. It has been reported that MAE efficiently extracted polyphenols (especially anthocyanin) from sour cherry peels, requiring less than 5 min [48]. Generally, MAE extraction is time-saving and energy-saving. A thermodynamic study indicated that DESs-based MAE was an efficient, endothermic, and spontaneous system for extracting polyphenols from sour cherry pomace [47]. Microwave power is a critical parameter that influences the extraction efficiency of polyphenols. Panic and co-workers revealed that the extraction of TAC from grape pomace increased with increasing microwave power [23]. Similar trends were observed for the extraction of polyphenols from mango peel [42], sour cherry peels [47], and onion skin [56]. As shown in Figure 5B, increasing microwave power (300, 400, and 500 W) steadily increased the yields of polyphenols from sour cherry peels [47].

### 4.3. Other or Combined Technologies

Other assisted techniques, such as infrared, PEF, and HVED, have intensified the extraction process. Rajha and co-workers extracted polyphenols from pomegranate peels using three assisted methods, namely conventional solid–liquid, ultrasound, and infrared [38]. Results showed that infrared obtained the utmost concentration of polyphenols (152 mg/g). This was ascribed to the high absorption of infrared radiation wavelengths by DESs and polyphenols. In addition, during the infrared extraction process, polyphenols were excited in different ways, such as stretching, bending, and twisting, and consequently, the extraction was improved. In order to increase the extraction yields of polyphenols from grapefruit peels [11] and pomegranate seeds [37], HVED was used as the assisted technology. Results showed that HVED significantly enhanced the diffusivity of polyphenols compared to the control. HVED may cause electrohydraulic discharges, accompanied by multiple secondary phenomena, including high-amplitude pressure shock waves, strong liquid turbulence, bubbles cavitation, UV radiations, and free radicals. These actions result in the disruption of complex structures and an increase in mass transfer, hence promoting extraction efficiencies. By jointly using PEF and DESs, the extraction efficiencies of rutin and quercetin in noni pomace were significantly higher than those of conventional organic extraction [52]. During PEF treatment, the electroporation of cell walls occurs when cells are subjected to an applied voltage with an associated electric field greater than the critical transmembrane potential, increasing the release of polyphenols.

Moreover, a combined strategy using ultrasound and microwave with NADESs has been developed to extract anthocyanins from grape pomace [23] and polyphenols from olive and grape pomace [24]. The extraction processes were carried out under microwave power of 300 W coupling with ultrasound power of 50 W for 10 min. The adopted simultaneous ultrasound/microwave-assisted extraction (SUMAE) achieved the highest anthocyanin yields with reduced energy consumption. This was attributed to the fact that double irradiation of ultrasound and microwave has synergistic effects on the extraction process: ultrasound ruptures the cells, and microwave promotes the release of polyphenols into DESs.

### 4.4. Comparison of These Assisted Technologies

Many studies reported that MAE was more efficient than UAE when coupling with DESs to extract polyphenols from agri-food by-products. For example, the extraction kinetic models of polyphenols from mulberry leaves confirmed that MAE (600 W, 60 °C, 20 min) was more efficient than UAE (250 W, 66 °C, 35 min) [36], MAE (500 W, 15 min) yielded more anthocyanin than UAE (500 W, 30 min) from blueberry peel [28], and MAE (180 W within 30 s) was more efficient than UAE (30 W, 50 °C) in the extraction of polyphenols from sour cherry pomace [48]. This may be ascribed to the fact that the power of applied microwave irradiation was higher than that of ultrasound, and microwave could reduce DESs viscosity more than ultrasound. In addition, HVED showed better performance than ultrasound in extracting polyphenols [37]. It has been reported that higher Zpolyphenol values were obtained via HVED than via ultrasound. In the applied ultrasound energy input (400–3600 kJ/kg), the polyphenol normalized content (Zpolyphenols) increased with the energy input. In the case of HVED energy input, Zpolyphenols values significantly increased in the range of 27–267 kJ/kg. Beyond 267 kJ/kg, higher HVED energy inputs resulted in lower Zpolyphenols, probably owing to the degradation of polyphenols. One possible reason is that high-energy HVED generates radical species that oxidize the extracted polyphenols.

## 5. Applications of Polyphenol-Rich DESs Extract in the Food, Cosmetic, and Pharmaceutical Industry

**Figure 6:**
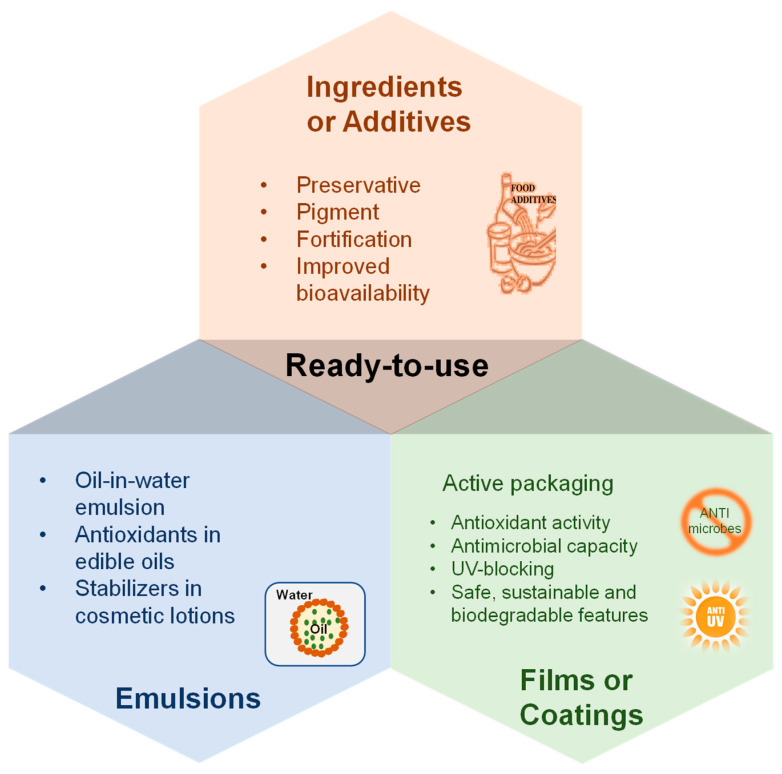
Applications of polyphenol-rich DESs extract in the food, cosmetic, and pharmaceutical industry.

DESs, particularly NADESs, are GRAS solvents and can be used in the food, cosmetic, and pharmaceutical industries [7,8]. NADESs are promising alternatives for producing biocompatible, ready-to-use extracts with specific biological activity without the need for extensive and costly downstream purification processes. The polyphenol-rich DESs extracts can be used as ready-to-use ingredients or additives for food products, cosmetic emulsions, and active packaging films/coatings (Figure 6).

### 5.1. Ready-to-Use Ingredients or Additives for Products

Recent research on polyphenol-rich extracts using NADESs revealed excellent antioxidant and antiproliferative activities and low cytotoxicity [19,24,33,91,97]. They play the role of preservatives, pigments, and fortifications. For example, NADESs extract from mango peel has been found to retard the oxidation of sunflower oil [42] and soybean crude oil [50] and increase the induction time, indicating its potential as a natural preservative in edible oils. Chocolate milk was fortified by a polyphenol-rich NADESs extract from cocoa by-products [91], and the electronic tongue result showed that the fortified chocolate milk had sensory acceptability within 10% of NADESs extract. Moreover, NADESs improved the bioavailability of polyphenol [24], anthocyanin [19,97], isoflavone [82], and rutin [98] in cells or rats compared to their aqueous solutions. Consequently, the polyphenol-rich NADESs extracts could serve as a delivery agent or administration vehicle in the pharmaceutical industry. Non-compartmental pharmacokinetic results showed that NADESs enhanced the bioavailability of anthocyanins by 140% compared to methanol:water:formic acid solvent [97]. By delaying gastric chyme neutralization, NADESs improved the stability of phenolic compounds during in vitro digestion. These findings imply that in addition to being an environmentally benign solvent for polyphenols extraction, NADESs can be employed as a ready-to-use vehicle for improving oral absorption of anthocyanins. As a concluding remark, the polyphenol-rich NADESs extracts are readily to be used as ingredients or additives for various products.

### 5.2. Food and Cosmetic Emulsions

Apart from the production of nutraceuticals or functional foods and pharmaceutical formulations, polyphenol-rich NADESs extract can also be used in cosmetic products. Polyphenol-rich NADESs extract can prevent lipid oxidation in oil-in-water (O/W) emulsions [77,81]. The Rancimat test showed that the presence of NADESs extract in the O/W emulsion increased the induction time and antimicrobial effect by 10-fold compared to that prepared with water [81]. Increasing the lipophilization of polyphenols leads to a positive change in their capacity to stabilize lipids against oxidation in O/W emulsions [77]. The incorporation of NADESs extract in O/W emulsion resulted in lower peroxide value and 2-thiobarbituric acid reactive substances, indicating its antioxidant action on lipids. The obtained stable O/W emulsions with DESs extract suggest its feasibility in food formulations. Based on the beneficial impact on keratinocyte growth, it is highly suggested that the polyphenol-rich NADESs extract can be readily used in the cosmetic industry [21,22]. Its use in cosmetic formulations brings at least three benefits: (1) protecting skin cells against oxidative stress and inflammation, (2) stimulating cell growth and regeneration, and (3) stabilizing products for longer shelf-life. Cosmetic emulsion with the addition of DESs extracts from tomato pomace showed satisfactory physicochemical characteristics [61]. NADESs could serve as a solvent to obtain a phenolic-rich extract that could be readily applicable to cosmetic formulations.

### 5.3. Active Packaging Films or Coatings

Additionally, the polyphenol-rich NADESs extract can be used to fabricate active packaging films or coatings for food applications (Table 7). NADESs function as plasticizer agents, improving the flexibility of film or coating. At the same time, the phenolic compounds in NADESs extract, such as anthocyanins, render films/coatings with multiple functional properties, including antioxidant, antimicrobial, UV-blocking, and pH-sensitive properties. For example, the incorporation of anthocyanin-rich NADESs extract into polyvinyl alcohol-based film reduced its glass transition temperature, transparency, and Young’s modulus, whereas it increased its water vapor permeability, elasticity, and water solubility [99]. The incorporation of polyphenol-rich NADESs extract into coatings achieved an in vitro antimicrobial activity of 72% against *Monilinia fructicola* [100]. These films and/or coatings containing polyphenol-rich DESs extracts can be used as pH indicators for evaluating food quality during storage [99] and active packaging to extend shelf-life [101,102,103]. For example, the chitosan/zein films containing *Rosa roxburghii* Tratt leaves extract showed better antioxidant and antibacterial activities, effectively inhibited the growth of foodborne pathogens, and extended the shelf lives of blueberries and fresh-cut cherry tomatoes [102]. Overall, these findings suggest that polyphenol-rich NADESs extract could be incorporated into films or coatings for active packing in the food industry.

## 6. Concluding Remarks and Future Perspectives

Polyphenols, which are ubiquitous in a huge variety of agri-food by-products, can provide plentiful beneficial effects for human health. This review timely reports the ongoing progress on the green extraction of polyphenols using DESs and assisted technologies. Polyphenol-rich agri-food by-product resources, extraction mechanisms, assisted technologies, and applications were highlighted. Concerning further research on the application of polyphenol-rich DESs extracts in the food, cosmetic and pharmaceutical industry, the following challenges should be addressed: (1) although many researchers have evidenced that polyphenol-rich DESs extracts is benign for humans due to its natural presence in various foods and the non-cytotoxicity of DESs, an in-depth eco-toxicological and cyto-toxicological profile is required; (2) when using polyphenol-rich DESs extract as a food ingredient or additive, the recommended daily intake (RDI) and its effect on sensory acceptance must be taken in account; (3) more research is needed to understand the mechanism underlying the extraction and biological activity of polyphenol-rich DESs extracts, such as the extraction efficiency, specificity, kinetics, thermodynamics, and the relationship between structure and bioactivity; and (4) elimination of the drawbacks of DESs (high viscosity and low vapor pressure) via assisted technologies to realize industrial application. In the near future, it is highly expected that polyphenol-rich DESs extracts will expand rapidly in industrial applications.

## Figures and Tables

**Table 1:** Some examples of recent applications of DESs and assisted techniques for extracting polyphenols from fruit by-products.

Fruit By-Products	Optimal DESs/NADESs	Value-Added Compounds	Assisted Techniques and Optimal Conditions	Yields (mg/g)	Reference
Apple pomace	ChCl:ethylene glycol (1:4)	Polyphenols (Procyanidin B2, chlorogenic acid, epicatechin hydrate, vanillin, and phloridzin)	~	~	[9]
	ChCl:glycerol (1:2)	Polyphenols (Quercetin, chlorogenic acid, gallic acid, phloretin, phloridizin, and rutin)	Ultrasound (acoustic intensity: 83.2 W/cm^2^, duty cycle: 75%)	TPC (GAE): 5.8	[10]
Citrus fruits industry by-products (orange, grapefruit, mandarin, lemon, pomelo peels)	Lactic acid:glucose (5:1)	Polyphenols (naringin)	HVED (Energy: 7.27~218 kJ/kg, 40 kV, 10 kA, 0.5 Hz, 160 J/pulse)	1.864	[11]
	ChCl:citric acid (1:1)	Hesperidin	~	112	[12]
	ChCl:levulinic acid:N-methyl urea (1:1.2:0.8)	Polymethoxylated flavonoids and glycosides of flavonoids	Ultrasound (200 W, 35 kHz)	18.8, 47.1	[13]
	ChCl:glycerol (1:3)	Polyphenols (quercetin, *p*-coumaric acid)	~	TPC (GAE): 24.2	[14]
	Lactic acid:glucose (5:1), L-proline:malic acid (1:1)	Polyphenols and flavonoids	~	TPC (GAE): 21.6, TFC (ECE): 0.97	[15]
	ChCl:ethylene glycol (1:4)	Polyphenols (*p*-coumaric acid and gallic acid)	~	TPC (GAE): 5.84	[16]
Grape solid wastes (stem, skin, seed, pomace, and lee)	Citric acid:maltose (4:1)	Anthocyanins	Ultrasound	TAC: 63.36	[17]
	ChCl:oxalic acid (1:2)	Polyphenols (catechin and quercetin-3-Oglucoside)	Microwave and ultrasound	~	[18]
	ChCl:malic acid (1:1)	Polyphenols and anthocyanins (malvidin-3-O-monoglucoside)	Ultrasound	TPC (GAE): 91, TAC (C3GE): 24	[19]
	ChCl:citric acid (1:2)	Flavan-3-ols, catechin, epicatechin, protocatechuic acid	Ultrasound (59 kHz)	TPC (GAE): 146.69	[20]
	Betaine:Glucose (1:1)	Polyphenols (flavan-3-ols)	Ultrasound (100 W)	~	[21]
	Betaine:citric acid (1:1)	Malvidin	~	56.66 µg/mL	[22]
	ChCl:citric acid (1:2)	Anthocyanins (malvidin-3-O-monoglucoside)	SUMAE (microwave 300 W + ultrasound 50 W)	TAC (M3GE): 1.77	[23]
		Polyphenols (anthocyanins, gallic acid, catechin and quercetin-3-O-glucoside)		TPC (GAE): 2.89	[24]
	ChCl:malic acid (1:1)	Anthocyanins (anthocyanin-3-O-monoglucosides and anthocyanin-3-(6-O-*p*-coumaroyl)monoglucosides)	Ultrasound (341.5 W, 37 kHz)	TAC (M3GE): 6.55	[25]
Blueberry processing wastes	ChCl:malic acid (1:1)	Anthocyanin	~	TAC (C3GE): 173	[26]
	ChCl:propylene glycol (1:2)	Anthocyanin	~	~	[27]
	ChCl:lactic acid (1:1)	Anthocyanins	Ultrasound and microwave (500 W, 15 min; 500 W, 20 kHz, 40 °C, 30 min)	TAC: 25.83 and 21.18	[28]
	ChCl:1,4-butanediol (1:3)	Polyphenols and Anthocyanins	Ultrasound (200 W, 63 °C, 20 min)	TPC (GAE): 41.6, TAC (C3GE): 11.40	[29]
	ChCl:oxlaic acid (1:1)	Anthocyanins	Ultrasound (325 W, 76 °C, 20 kHz, 3.2 min)	TAC (C3GE): 24.3	[30]
	ChCl:1,4-butanediol (1:3)	Anthocyanins (cyanidin-3-rutinoside)	Ultrasound (380 W, 37 kHz, 55 °C, 40 min)	TAC (C3GE): 9.32	[31]
Cranberry, strawberry, and raspberry extrudate waste	ChCl:betaine hydrochloride: levulinic acid (1:1:2)	Procyanidins and anthocyanins	Ultrasound	TAC: 1.30	[32]
	Citric acid:betaine (1:1)	Ellagic Acid	Ultrasound	TPC (GAE): 44.8	[33]
	ChCl:glycolic acid:oxalic acid (1:1.7:0.3)	Anthocyanins	~	~	[34]
Mulberry (*Morus alba* L.) leaves	ChCl:citric acid (2:1)	Chlorogenic acid	Ultrasound (600 W, 25 kHz)	22.7	[35]
	ChCl:glycerol (1:2)	Neochlorogenic acid, chlorogenic acid, cryptochlorogenic acid, caffeic acid, rutin, isoquercetin, astragalin	Microwave (600 W)	0.240, 4.51, 0.342, 0.286, 1.47, 0.971 and 0.538	[36]
Pomegranate waste (peel, mesocarp, and seeds)	ChCl:acetic acid (1:1)	Polyphenols (mainly ellagitannins)	HVED (Energy: 7.27~218 kJ/kg, 40 kV, 10 kA, 0.5 Hz, 160 J/pulse, electrical pulses: 10~800)	TPC (GAE): 9.5	[37]
	Eight different DESs	Polyphenols (tannin)	Infrared (70~170 W)	TPC (GAE): 152	[38]
	ChCl:urea (1:2)	Punicalagin and ellagic acid	Ultrasound (120 W and 300 W, 20 min),	130.65 and 2.04	[39]
	ChCl:lactic acid (1:1)	Polyphenols	Ultrasound	TPC (GAE): 6.4 mg/mL	[40]
	ChCl:glycerol (1:11)	Polyphenols and flavonoids	~	TPC (GAE): 273, TFC (QE): 20.1	[41]
Mango peel	Sodium acetate:lactic acid (1:3)	Polyphenols (mangiferin)	Microwave (436.45 W)	TPC (GAE): 56.17	[42]
Mangosteen peel	ChCl:lactic acid (1:2)	Anthocyanins	Ultrasound (amplitude: 60%)	TAC (ECE): 2.38	[43]
Pear industrial by-products	Lactic acid:glucose (5:1)	Gallic acid and rutin	Ultrasound (20 kHz, 200 W)	~	[44]
	ChCl:glutaric acid (1:1)	Rutin, hyperoside, and isoquercitrin	~	~	[45]
Peach peels	Lactic acid:glycerol (1:1)	Polyphenols and chlorogenic acid)	~	TPC (GAE): 10.5 and 2.70	[46]
Sour cherry pomace	Citric acid:ethylene glycol (1:4)	Polyphenols	Microwave (500 W)	TPC (GAE): 33.61	[47]
	ChCl:malic acid (1:1)	Neochlorogenic acid	Ultrasound and microwave (37 kHz, 30 W, 50 °C; 180 W within 30 s)	0.145 and 0.178	[48]
Sea buckthorn leaves	ChCl:1,4-butanediol (1:3)	Rutin, quercetin-3-O-glucoside, quercetin, kaempferol, isorhamnetin	Microwave (600 W)	8.99, 1.81, 9.11, 0.45, 0.49	[49]
*Annona muricata* L. Leaves	Linium chloride:glycerol (1:2), Linium chloride:xylitol (1:2)	Rutin and catechin	Ultrasound	~	[50]
Date palm seeds	ChCl:formic acid (1:2)	Polyphenols	Microwave (400 W)	TPC (GAE): 128	[51]
Noni-processing waste	ChCl:oxalic acid (1:2)	Rutin and quercetin	PEF (field strength: 0.64–1.84 kV/cm), number of pulses: 25–125)	16.21 and 19.85	[52]

Note: C3GE: cyanidin-3-O-glucoside equivalent, ChCl: choline chloride, ECE: epicatechin equivalent, GAE: gallic acid equivalent, HVED: high-voltage electric discharge, PEF: pulsed electric field, QE: quercetin equivalent, SUMAE: simultaneous ultrasound/microwave-assisted extraction, TAC: total anthocyanins content, TFC: total flavonoid content, and TPC: total phenolic content.

**Table 2:** Some examples of recent applications of DESs and assisted techniques for extracting polyphenols from vegetable by-products.

Vegetable By-Products	Optimal DESs/NADESs	Value-Added Compounds	Assisted Techniques and Optimal Conditions	Yields (mg/g)	Reference
Onion solid wastes	ChCl:urea: H_2_O (1:2:4)	Polyphenols (Quercetin, kaempferol, and myricetin)	Microwave (100 W)	TPC (GAE): 80.5	[55]
	ChCl:urea (1:2)		Microwave (300 W)	TPC (GAE): 223	[59]
	Temperature-responsive DESs	Flavonoids (Quercetin, kaempferol, luteolin, and quercetin-3-O-β-D-glucoside)	Microwave (554 W)	47.83	[56]
	ChCl:urea (1:1)	Tannic acid	Ultrasound (Duty cycle: 10%, amplitude: 13%, 26 W, intensity 20.47 w/cm^2^, 20 kHz)	1.71	[57]
	Sodium propionate-based DESs	Polyphenol (quercetin 4′-O-glucoside and quercetin) and flavonoids	~	TPC (CAE): 137.5, TPC (RtE): 136.5	[58]
	Betaine:glycolic acid (1:2)	Quercetin	Ultrasound	14.79 µg/mL	[60]
Tomato industry by-products	Lactic acid:glucose (5:1)	Polyphenols (caffeic acid, naringenin, catechin, quercetin, rutin)	Ultrasound (20 kHz, 200 W)	~	[44]
	ChCl:lactic acid (1:2)	Phenolic acids (chlorogenic acid)	~	52.3	[61]
Violet potato peels	ChCl:lactic acid	Polyphenols	Microwave and ultrasound	~	[62]
Kale waste	Betaine:glycerol (1:3)	Polyphenols	~	TPC (GAE): 16.8	[63]
Bitter melon leaves	ChCl:acetic acid (1:2)	Polyphenols (gallic acid, chlorogenic acid, vanillic acid, quercetin-3-glucoside) and flavonoid (epicatechin)	Ultrasound	TPC (GAE): 82.1, TFC (ECE): 1.26	[64]
Pepper leaves	ChCl:glucose (1:1)	Polyphenols (gallic, protocatechuic, chlorogenic, cinnamic, and coumaric acids)	Ultrasound (42 kHz)	TPC (GAE): 22.4	[65]
Lotus leaf	Lactic acid:glycerol (1:2)	Flavonoids (quercetin 3-O-glucoside)	~	TPC (GAE): 113, TFC (RtE): 126	[66]

Note: CAE: caffeic acid equivalent, ChCl: choline chloride, ECE: epicatechin equivalent, GAE: gallic acid equivalent, RtE: rutin equivalent, TFC: total flavonoid content, and TPC: total phenolic content.

**Table 3:** Some examples of recent applications of DESs and assisted techniques for extracting polyphenols from tree nut by-products.

Tree Nut By-Products	Optimal DESs/NADESs	Value-Added Compounds	Assisted Techniques and Optimal Conditions	Yields (mg/g)	Reference
Chestnut shell	ChCl:oxalic acid (1:1)	Polyphenols (gallic acid, ellagic acid, catechin hydrate, and procyanidin B2), total condensed tannin	Microwave	TPC (GAE): 295, total condensed tannins: 229.6	[67]
	ChCl:n-propanol (1:1)	Ellagic acid	Ultrasound	4.64	[68]
*Carya cathayensis* Sarg peel	ChCl:malic acid (1:1)	Polyphenols (catechin, procyanidin B1, 2,3-dihydroxybenzoic acid, pinocembrin, procyanidin B3, and myricetrin) and flavonoids	Pulse ultrasound (20 kHz, 400 W)	TPC (GAE): 60.8	[69]
				TPC (GAE): 76.3, TFC (QE): 793	[70]
Hazelnut pomace	ChCl:1,2-propylene glycol (1:4)	Polyphenols (quinic acid, gallic acid, catechin, protocatechuic acid, and quercetin-3-O-rhamnoside)	Microwave	~	[71]

Note: ChCl: choline chloride, GAE: gallic acid equivalent, QE: quercetin equivalent, TFC: total flavonoid content, and TPC: total phenolic content.

**Table 4:** Some examples of recent applications of DESs and assisted techniques for extracting polyphenols from cereal by-products.

Cereal By-products	Optimal DESs/NADESs	Value-Added Compounds	Assisted Techniques and Optimal Conditions	Yields (mg/g)	Reference
Wheat bran	ChCl:glycerol (1:3)	Polyphenols	Ultrasound (37 KHz; 140 W; Power density: 35 W/L)	TPC (FAE): 17.78, TFC (RtE): 7.27	[73]
Brewer’s spent grain	ChCl:glycerol (1:2)	Polyphenols (ferulic and coumaric acids)	Microwave	TPC (GAE): 2.30	[72]
Black rice bran	Lactic acid:fructose (5:1)	Anthocyanin	Ultrasound (amplitude: 21.31%)	TAC (C3GE): 109 mg/L	[74]
Tartary buckwheat hull	ChCl:glycerol (1:1)	Rutin	Ultrasound (200 W, 20 kHz)	9.5	[75]
Red rice bran	ChCl:xylitol (1:1)	Polyphenols	~	~	[77]
Foxtail millet bran	Betaine:glycerol (1:2)	Polyphenols and flavonoids (coumaric acid, apigenin-C-dihexoside, and coumaroylspermidine)	Ultrasound (247 W)	TPC (FAE): 7.80	[76]

Note: C3GE: cyanidin-3-O-glucoside equivalent, ChCl: choline chloride, FAE: ferulic acid equivalent, GAE: gallic acid equivalent, RtE: rutin equivalent, TAC: total anthocyanins content, TFC: total flavonoid content, and TPC: total phenolic content.

**Table 5:** Some examples of recent applications of DESs and assisted techniques for extracting polyphenols from oil-bearing crop by-products.

Oil-Bearing Crop By-products	Optimal DESs/NADESs	Value-Added Compounds	Assisted Techniques and Optimal Conditions	Yields (mg/g)	Reference
Olive oil industry by-products	Glycerol:glycine: water (7:1:3)	Oleuropein, flavone glycosides	~	TPC (GAE): 106	[84]
	ChCl:citric acid (1:2)	Polyphenols (Oleuropein, hydroxytyrsol)	Microwave (200 W) and ultrasound (60 kHz, 280 W)	TPC (GAE): 34.1 (12.9, 3.37)	[80]
	ChCl:citric acid (1:1), ChCl:ethylene glycol (1:2)	Hydroxytyrosol, tyrosol, and oleuropein	Ultrasound (40 KHz)	~	[89]
	Citric acid:glycine:water (2:1:1)	Hydroxytyrosol	~	~	[87]
	ChCl:acetic acid (1:2), ChCl:malic acid (1:1), ChCl:malonic acid (1:1), and ChCl:citric acid (2:1)	Ferulic acid, kaempferol, luteolin, oleuropein, tyrosol	~	TPC (GAE): 34.6	[86]
		Polyphenols	~	TPC (GAE): 19.8	[81]
	ChCl:fructose:water (5:2:5)	Oleuropein, caffeic acid	Ultrasound (37 KHz; 140 W; Power density: 35 W/L)	1.63 and 0.113	[85]
	ChCl:glycerol (1:5)	Luteolin-7-oglucoside, lleuropein, 3-hydroxytyrosol, rutin, and luteolin	~	~	[88]
Soy molasses	ChCl:citric acid (1:2)	Isoflavone (daidzein, genistein and puerarin)	Ultrasound (37 KHz; 100 W)	~, 2.49, 7.23	[82]
Kudzu roots				12.13, 2.51, 0.37	
Peanut hull	Four Imidazole-based DESs (1:1)	Quercetin	Microwave (50 kHz)	0.43	[79]
Sunflower disks	ChCl:1,4-butanediol (1:2)	Chlorogenic acids	Ultrasound (40 KHz; 300 W; Power density: 0.5 W/cm^2^)	6.16	[78]

Note: ChCl: choline chloride, GAE: gallic acid equivalent, and TPC: total phenolic content.

**Table 6:** Some examples of recent applications of DESs and assisted techniques for extracting polyphenols from other agri-food by-products.

Other Agri-Food By-Products	Optimal DESs/NADESs	Value-Added Compounds	Assisted Techniques and Optimal Conditions	Yields (mg/g)	Reference
Coffee and cocoa industry residues	Fourteen hydrophilic and hydrophobic DESs	Chlorogenic acid	~	~	[90]
	ChCl:citric acid (2:1), ChCl:glycerol (1:2), ChCl:glucose (1:1), betaine:citric acid (1:1), betaine:glycerol (1:2) and betaine:glucose (1:1)	Polyphenols	Ultrasound (150 W)	TPC (GAE): 20.3, 19.2, 21.6, 18.2, 15.3, 22.8	[91]
	ChCl:lactic acid (1:2)	Polyphenols and chlorogenic acid	~	TPC (GAE): 52.9	[92]
	Betaine:triethylene glycol (1:2)	Chlorogenic acid	Ultrasound (200 W, 37 kHz)	4.64	[93]
	ChCl:1,2-propanediol (1:2)	Gallic acid	~	138	[94]
Spent hops	ChCl:ethylene glycol (1:2), ChCl:propylene glycol (1:2), ChCl:glycerol (1:2), ChCl:lactic acid (1:2)	Xanthohumol	~	0.42–0.75	[95]

Note: ChCl: choline chloride, GAE: gallic acid equivalent, and TPC: total phenolic content.

**Table 7:** Films or coatings containing polyphenol-rich NADESs extracts for food packaging applications.

Polyphenol-Rich NADESs Extracts			Film Matrix	Effects on Film Properties	Purpose	Reference
Main Compounds	By-Product	NADESs				
Polyphenols and flavonoids	Pomegranate peel	ChCl:glycerol	Chitosan	Reduced water sorption and shade color of films	Edible packaging film	[41]
Anthocyanin	Black rice bran	Lactic acid:fructose	Polyvinyl alcohol	Reduced the glass transition temperature, transparency, and young modulus, whereas increased water vapor permeability, elasticity, and water solubility.	Edible pH indicators for evaluating food quality during storage	[99]
Cinnamic acid, nordihidroguiaretic acid, and quercetin	*Larrea divaricata*	Lactic acid:glucose:water	Pectin	Achieved an in vitro antimicrobial activity of 72% against *Monilinia fructicola*.	Bioactive coating	[100]
Phenolic compounds	*Moringa oleifera* leaves	ChCl:glycerol, ChCl:lactic acid	Methylcellulose	All films are uniform, clear, and transparent with smooth, homogeneous surfaces.	Packaging films for sliced wheat bread	[101]
Ellagic acid, quercitrin, isoquercitrin	*Rosa roxburghii* Tratt leaves	ChCl:lactic acid	Chitosan/zein	Improved the mechanical properties, light barrier properties, water vapor permeability, antioxidant, and antibacterial activities of the chitosan/zein films.	Active packaging for fruit preservation	[102]
Catechin, protocatechuic acid ethyl ester, benzoic acid, syringic acid, and vanillin	Date palm leaves	ChCl:glycerol	Cellulose nanoparticles and soy protein isolate	Significantly increased antioxidant and antibacterial properties of films.	Active packaging films	[103]

## Data Availability

Not applicable.

## Abbreviations

ANN: artificial neural network, C3GE: cyanidin-3-O-glucoside equivalent, CAE: caffeic acid equivalent, ChCl: choline chloride, COSMO-RS: Conductor-like Screening Model for Real Solvents, DESs: deep eutectic solvents, ECE: epicatechin equivalent, FAE: ferulic acid equivalent, FAO: Food and Agricultural Organization, GAE: gallic acid equivalent, GRAS: generally recognized as safe, HBAs: hydrogen bond acceptors, HBDs: hydrogen bond donors, HVED: high-voltage electric discharge, HVEDAE: high voltage electric discharge assisted extraction, M3GE: malvidin-3-glucoside equivalent, MAE: microwave-assisted extraction, NADESs: natural deep eutectic solvents, O/W: oil-in-water, PEF: pulsed electric field, RSM: response surface methodology, RtE: rutin equivalent, SDGs: Sustainable Development Goals, SEM: scanning electron microscope, SLE: solid-liquid extraction, SUMAE: simultaneous ultrasound/microwave-assisted extraction, TAC: total anthocyanin content, TFC: total flavonoid content, TPC: total phenolic content, UAE: ultrasound-assisted extraction.
